# Characterization of the complete chloroplast genome of *Lycium barbarum* (Solanales: Solanaceae), a unique economic plant to China

**DOI:** 10.1080/23802359.2018.1509930

**Published:** 2018-10-03

**Authors:** Guolun Jia, Guiliang Xin, Xiaolong Ren, Xiaomin Du, Huidong Liu, Nan Hao, Cuiping Wang, Xilu Ni, Wenzhe Liu

**Affiliations:** a Key Laboratory of Resource Biology and Biotechnology in Western China (Northwest University), Ministry of Education, School of Life Science, Northwest University, Xi’an, China;; b State Key Laboratory of Seeding Bioengineering, Ningxia Forestry Institute, Yinchuan, China

**Keywords:** Chloroplast genome, Illumina sequencing, phylogenetic analysis, *Lycium barbarum*, Solanaceae

## Abstract

*Lycium barbarum* (Solanaceae) is a unique economic plant to China. The complete chloroplast (cp)genome was sequenced and assembled by using Illumina paired-end reads data. The circular cp genome is 155,656 bp in size, including a pair of inverted repeat (IR) regions of 25,451 bp, a large single copy (LSC) region of 86,554 bp and a small single copy (SSC) region of 18,200 bp. Besides, 15 genes possess a single intron, while another three genes (clpP, rps12 and ycf3) have a couple of introns. The GC content of entire *L. barbarum* cp genome, LSC, SSC and IR regions are 37.8%, 35.9%, 32.3%, and 43.2%, respectively. Phylogenetic analysis based on the concatenated coding sequences of cp PCGs showed that *L. barbarum* and *Atropa belladonna* are closely related with each other within the family Solanaceae.

*Lycium barbarum* L., belonging to the family Solanaceae, is a perennial deciduous shrub (Kuang and Lu 1978), unique economic plant (Brewbaker 1959) and valuable traditional medicinal plant to China. It grows mainly in the arid and semi-arid regions of Ningxia, Xinjiang, inner Mongolia and so on (Kuang and Lu 1978). Previous studies had shown that the root and aerial parts of the plant have commonly been used in traditional medicine, which was recorded as a famous and historic drug for its effects of improving liver and kidney function, promoting body immunity, anti-aging, profiting lung and eyesight. Previous phylogeny studies of Solanaceae plants were mainly based on a few chloroplast genes or ITS (Olmstead et al. 2008; Hajrasouliha et al. 2014), however, genome information of *L. barbarum* has been poorly studied. Considering the lack of complete chloroplast (cp) genome sequence data of *L. barbarum*, this study will be useful for the research on the phylogenetic relationships of *L. barbarum* and Solanaceae.

Fresh leaves of *L. barbarum* were collected in Lycium germplasms nursery, Ningxia Forestry Institute (38°28′N, 106°16′E; Ningxia, NW China). Total genomic DNA was extracted with the modified CTAB method (Doyle JJ and Doyle JL 1987) to construct a shotgun library which was used for next-generation sequencing on the Illumina Hiseq 2500 Sequencing System (Illumina, CA, USA). We assembled cp genome using the program MITObim v1.8 (Hahn et al. 2013), with that of *Solanum tuberosum* (GenBank: JF772171) as the initial reference. The map of the complete cp genome was generated using the web-based tool OGDRaw v1.2 (http://ogdraw.mpimp-golm.mpg.de/) (Lohse et al. 2013). The complete cp genome sequence has been submitted to GenBank under the accession number MH032560.

The complete cp genome of *L. barbarum* is a circular and double-stranded DNA molecule of 155,656 bp in length with a typical quadripartite structure, containing two inverted repeat (IR) regions of 25,451 bp separated by a large single copy (LSC) region of 86,554 bp and a small single copy (SSC) region of 18, 200 bp. It encodes 132 complete genes, including 87 protein-coding genes, 37 transfer RNA genes and eight ribosomal RNA genes. In addition, 9 PCG genes (*atpF, ndhA, ndhB, rps16, rpl2, rpl16, rpoC1, petD* and *petB*) and 6 tRNA genes (*trnA-UGC, trnG-UCC, trnI-GAU, trnK-UUU, trnL-UAA* and *trnV-UAC*) harbor a single intron, while three other genes (*clpP, rps12* and *ycf3*) possess two introns. The overall GC content of *L. barbarum* cp genome is 37.8%, while the corresponding values of the LSC, SSC and IR regions are 35.9%, 32.3% and 43.2%, respectively.

To investigate the phylogenetic position of *L. barbarum*, a neighbor-joining (NJ) phylogenetic tree (Figure 1) was made based on the concatenated coding sequences of cp PCGs for 18 plastid genomes from published species of Solanaceae using MEGA7 with 1000 bootstrap replicates (Kumar et al. 2016) (http://www.megasoftware.net/). The result of the phylogenetic analysis shows that *L. barbarum* is closely related to the species of *Atropa belladonna*. The complete cp genome sequence adds valuable information for the study of the genetic diversity of *L. barbarum* and Solanaceae.

**Figure 1.**
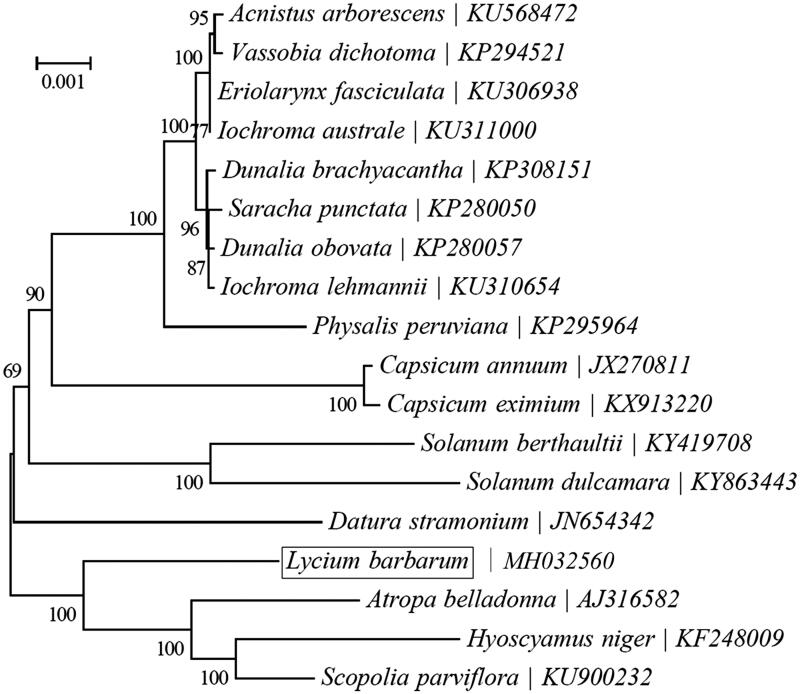
Phylogeny of 13 species within the order Solanaceae based on the neighbor-joining (NJ) analysis of chloroplast PCGs.
